# Impact of Multi-leaf Collimator Parameters on Head and Neck Plan Quality and Delivery: A Comparison between Halcyon™ and Truebeam® Treatment Delivery Systems

**DOI:** 10.7759/cureus.3648

**Published:** 2018-11-28

**Authors:** Taoran Li, Ryan Scheuermann, Alexander Lin, Boon-Keng Kevin Teo, Wei Zou, Samuel Swisher-McClure, Michelle Alonso-Basanta, John N Lukens, Alireza Fotouhi Ghiam, Chris Kennedy, Michele M Kim, Dimitris Mihailidis, James M Metz, Lei Dong

**Affiliations:** 1 Department of Radiation Oncology, Perelman School of Medicine, University of Pennsylvania, Philadelphia, USA

**Keywords:** halcyon, multi-leaf collimator, head and neck, radiation therapy, fff, vmat, imrt

## Abstract

Purpose

A new dual-layer multi-leaf collimator (MLC) system with several improved characteristics was introduced with the Varian Halcyon™ treatment platform. This study evaluated this new MLC’s impact on head and neck plan quality and delivery efficiency.

Methods

Nine patients were retrospectively studied with Institutional Review Board (IRB) approval. To compare plan quality between the Halcyon dual-layer MLC and Truebeam® MLC, all patients were replanned with the same prescription and target coverage following the institutional clinical protocol for both platforms and using both intensity modulated radiation therapy (IMRT) or volumetrically modulated arc therapy (VMAT) techniques. Organs-at-risk (OAR) dose-volume histogram (DVH) statistics were compared along with total plan monitor units (MU). To evaluate delivery efficiency, actual beam-on time for five patients’ plans were recorded and compared. To evaluate the impact of MLC performance parameters on plan quality, virtual MLC models were generated by matching Truebeam MLC’s parameters to those of the Halcyon dual-layer MLC both individually and combined. OAR doses were then compared between these virtual MLCs, the Truebeam MLC, and the actual Halcyon MLC.

Results

Overall the Halcyon dual-layer MLC provided similar plan quality compared to Truebeam MLC for VMAT plans, and improved sparing for majority of the OARs when using IMRT. Paired comparison showed median dose differences in mean doses to the parotids, cochlea, esophagus, and larynx ranged from -0.83 Gy to 0.37 Gy for VMAT, and from -4.79 Gy to -0.04 Gy for IMRT, with negative values indicating improved performance by Halcyon. Despite a slight increase in plan MU, the Halcyon reduced the total beam-on time by 42.8 ± 8.5%. Virtual MLC simulations demonstrated that matching MLC transmission accounted for nearly half of the total dose difference between Halcyon and Truebeam IMRT plans.

Conclusion

When compared to the Truebeam, the Halcyon’s dual-layer MLC achieved similar plan quality using VMAT, and improved OAR sparing using IMRT, while providing nearly twice as fast treatment delivery. Reduction in MLC transmission is the dominating factor contributing to dosimetric differences in OAR sparing.

## Introduction

Radiation therapy plays a key role in managing head and neck cancer. Due to the complexity of the shape and configuration of tumor and lymph nodes in the target region, and close proximity of organs-at-risk (OARs), intensity-modulated radiation therapy (IMRT) or volumetrically modulated arc therapy (VMAT) has been widely used in treatment planning for head neck patients.

The delivery of IMRT/VMAT treatment plans relies heavily on the multi-leaf collimator (MLC). Therefore, it is expected that MLC parameters such as inter-leaf leakage, leaf transmission, dosimetric leaf gap (DLG), leaf width, leaf over-travel limits, and leaf speed, play important roles in impacting the quality of the treatment plan. Previous studies have heavily investigated the impact of MLC leaf width on the quality of IMRT plans. Burmeister et al. [1] and Wu et al. [2] investigated the difference in plan quality between 1 cm, 0.5 cm, and 0.25 cm leaf widths for head and neck and central nervous system (CNS) malignancies, and concluded that leaf width has limited impact on plan quality for large volume targets. This conclusion was also supported by several other studies [3-8]. Topolnjak et al. investigated the impact of MLC characteristics on plan quality for seven head and neck cancer patients using a hypothetical linac model, and concluded that leaf transmission has a major impact on the normal tissue mean dose [9]. In their work the increase of leaf transmission from 0.75% to 1.5% resulted in an increase in parotids mean dose by 1.8 Gy. The impact of other MLC parameters such as DLG and leaf speed has not been extensively tested or reported for head and neck applications.

Recently a new straight-through jawless treatment delivery system was introduced by Varian Medical Systems (Palo Alto, CA) trademarked Halcyon™. In this system, a new dual-layer MLC design was introduced to fulfill jawless configuration while simultaneously providing sufficient beam attenuation and shape modulation. The new MLC design, referred to here as “dual-layer MLC,” features a dual-layer of stacked and staggered MLC leaves with 1 cm leaf width in each layer when projected to the isocenter plane, which is at 100 cm source-to-axis distance (SAD). This MLC system also features faster speed than Varian’s current mainstream MLC system used in the Truebeam® accelerator product lines, the Millennium 120, referred to here as TB MLC, providing 5.0 cm/s movement at the isocenter plane compared to 2.5 cm/s for the Millennium 120. The Halcyon dual-layer MLC can also achieve full-field modulation with 100% leave over-travel and interdigitation. The Halcyon linac system offers only the six megavoltage (6 MV) flattening-filter-free (6FFF) mode for treatment delivery. The omission of flattening filter increases the beam output and has a lower average energy than 6 MV beams produced with the same maximal energy but flattened with a filter (6X). This lower average energy, combined with the improvement in the MLC design, provided a significant improvement in the leakage and transmission properties of the dual-layer MLC compared to Millennium 120 MLC: 0.7% per layer leaf transmission for the dual-layer MLC compared to around 1.5% leaf transmission for Millennium 120 used in the Truebeam linac. Dosimetric leaf gap for this dual-layer MLC was changed to 0.1 mm in the Halcyon beam model. At the time of this study, the dual-layer MLC is only able to provide modulation using the distal layer of MLC, i.e., the layer away from the radiation source and closer to the patient, while the proximal MLC layer follows the opening of distal layer MLC, providing additional shielding essentially similar to backup jaws but for each individual interleaf region. Therefore, in this study the effective leaf width for all Halcyon plans is 1.0 cm, compared to 0.5 cm for the regular Truebeam Millennium 120 MLC design. In the recently released Halcyon 2.0 both layers of MLC participate in active fluence modulation, for which results were still being generated and not included in this study.

Since the dual-layer MLC has improved transmission, speed, over-travel, and DLG, but twice as large leaf width, it is important to understand the impact to plan quality and delivery efficiency when compared to the regular Truebeam Millennium 120 MLC design. In the first part of this study, we compared the dosimetric and delivery parameters for plans generated with both types of delivery systems to determine if there were any significant differences between plans generated with the two MLC designs. In the second part of the study, we focused on the cases with significant difference, and studied how these MLC parameters contributed to those differences.

## Materials and methods

Nine head and neck cancer patients previously treated at our institution were included in this study under an Institutional Review Board (IRB)-approved retrospective study protocol.

Overall plan quality evaluation

All nine cases were planned in parallel using combinations of the Truebeam with Millennium 120 (TB MLC) and the Halcyon system with the dual-layer MLC system as well as two planning techniques: IMRT and VMAT. For the beam arrangements, all cases used nine equally spaced beams for IMRT and two full or half arcs for VMAT. Half arcs were only used for two unilateral post-operative cases to reduce dose spread to the contralateral neck, which is our standard institutional approach in such patients. The same isocenter was used across all different planning techniques and platforms. Plans were generated by experienced physicists and dosimetrists and subsequently reviewed by a single attending physician to ensure the quality of the plans meet clinical requirements. To simulate how a clinical plan would be generated, 6X was used for Truebeam and 6FFF for Halcyon, which is our standard beam energy choice in our clinical planning procedure. The planners were instructed to approach each plan as a separate clinical case and to achieve overall the best plan without comparing between platforms. All plans were performed with the Eclipse™ treatment planning systems using Photon Optimizer™ and a pre-clinical Anisotropic Analytical Algorithm (AAA) v15.1 dose calculation algorithm with the same dose calculation grid size (2.5 mm). All plans for the same patients were normalized so that the same dose coverage (100% Rx dose covering 95% of PTV volume) was achieved for the highest prescribed target.

Key dosimetric parameters of clinical interest were compared among different planning techniques and delivery systems: global maximal dose, maximum cord dose, and mean dose to the parotids, cochleae, esophagus, and larynx (non-target). Total monitor units required to delivery treatment were also compared. The paired treatment plans, i.e. plans created with different delivery system for the same patient, were compared statistically with the Wilcoxon signed-rank test. This comparison was done separately for IMRT and VMAT. Statistical significances (p-values) from this test were reported.

Plan delivery time comparison

One of the advantages of the Halcyon platform is that the gantry rotation speed is four times that of the Truebeam linac. This potentially enables plans to be delivered in a much shorter time and therefore increases efficiency and patient throughput. In this study the treatment delivery efficiency was evaluated by comparing the actual total beam-on time between the Truebeam and Halcyon platforms and between IMRT and VMAT plans. Five patients’ plans were included in this part of the study, each with four plans delivered: Truebeam-IMRT, Truebeam-VMAT, Halcyon-IMRT, Halcyon-VMAT. All plans were delivered with automation enabled, i.e. the machine automatically delivered all treatment fields without operator intervention, to mimic actual clinical usage.

Impact of MLC parameters on plan quality

An additional study was completed to identify the MLC parameters that contributed most to the observed differences in the plan quality. Because the Halcyon platform does not allow end users to modify the beam data within the treatment planning system, the TB MLC’s transmission, leaf speed, and DLG parameters were altered in the TB beam model configuration to (virtually) match with those of the Halcyon dual-layer MLC, as shown in (Table 1). It should be noted that these values are based on pre-clinical system, and might be different from the clinical system. In order to isolate only the effect from selected MLC parameters on plan quality, the optimization objective settings were extracted from clinical plans and were kept the same for all plans for the same patient case. All plans using different simulated TB MLC and actual dual-layer MLC were optimized anew using the same objectives from the plan done with original TB MLC parameters, 200 iterations, and automatic intermediate dose calculation. All re-optimized plans were normalized to 95% PTV covered by 100% prescription dose. The re-optimized plans, now with different hypothetical MLC systems, were then compared to the Halcyon dual-layer MLC plans in terms of dosimetric difference. In this way, the change or combination of changes to the TB MLC parameters that yielded the closest dosimetric parameters to the dual-layer MLC plan represents the parameters that contributed the most to the difference in plan quality between two different delivery systems. The comparison baselines were set to plans generated using Truebeam 6FFF photons and Millennium 120 MLC since Halcyon only has 6FFF energy. Because of the extensive amount of planning needed to be done for all hypothetical MLC types, this study was only performed for three patients’ IMRT plans that exhibited relatively large differences in the previous planning comparison between TB and dual-layer MLCs.

**Table 1:** Simulated Truebeam® multi-leaf collimator (TB MLC) with different parameters from the Halcyon™ dual-layer MLC. The parameters inside the bracket are simulated hypothetical MLC parameters, including leaf width, dosimetric leaf gap (DLG), leaf speed, and transmission. Values used here were based on pre-clinical system and might be different from clinical system.

	Leaf Width	DLG	Leaf Speed	Transmission
TB MLC DLG Matched	0.5 cm	(0.01 mm)	2.5 cm/s	1.5%
TB MLC Leaf Speed Matched	0.5 cm	0.13 mm	(5.0 cm/s)	1.5%
TB MLC Transmission Matched	0.5 cm	0.13 mm	2.5 cm/s	(0.7%)
TB MLC All 3 Matched	0.5 cm	(0.01 mm)	(5.0 cm/s)	(0.7%)
Halcyon Dual-Layer MLC	1.0 cm	0.01 mm	5.0 cm/s	0.7%

## Results

Plan comparisons

**Figure 1:**
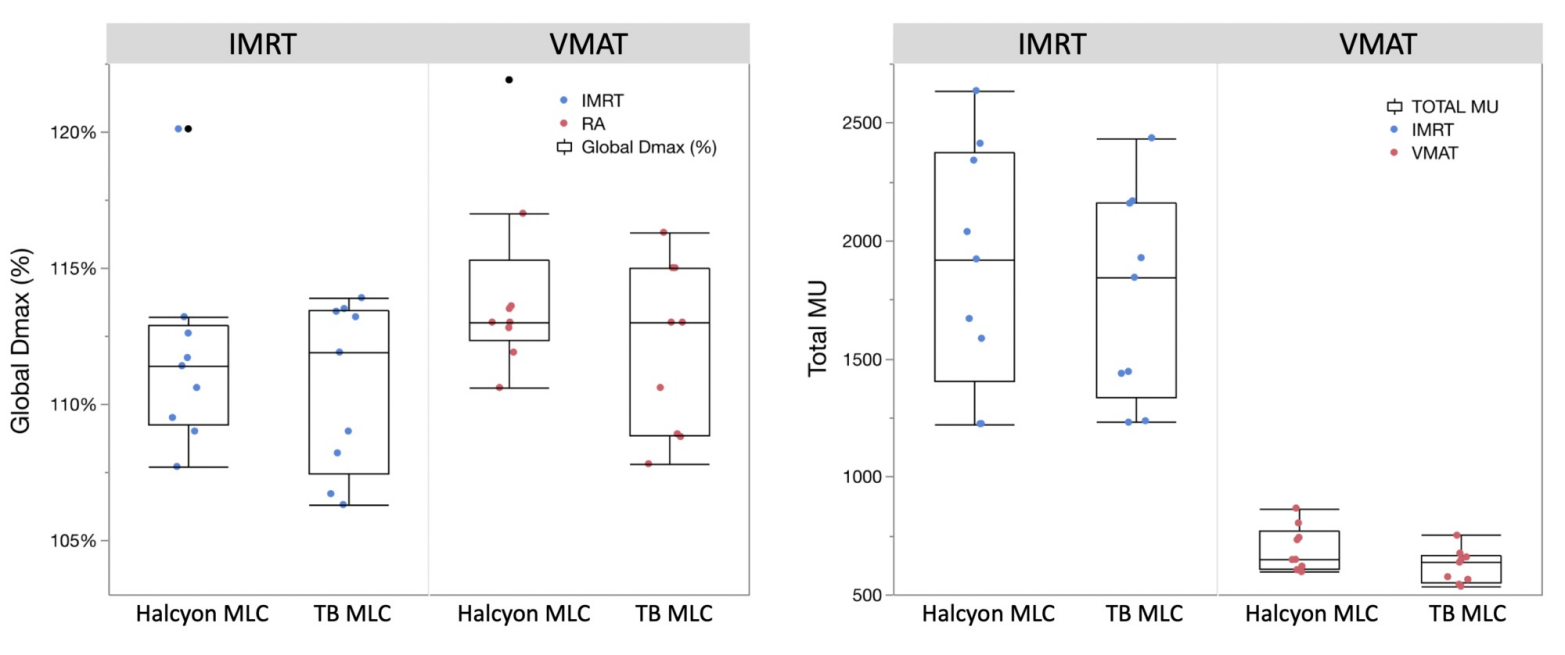
Box plots and individual data point comparison of global maximal dose in % (left) and total number of monitor units (MU) (right) across two multi-leaf collimator (MLC) types and two planning techniques. IMRT: Intensity-modulated radiation therapy; VMAT: Volumetrically modulated arc therapy; MU: Monitor unit.

It is meaningful to compare global maximal dose (Dmax) under the same target coverage since all plans were normalized to have the same target coverage by the Rx dose. The left pane in Figure 1 compares global Dmax across IMRT/VMAT and dual-layer/TB MLCs. The horizontal line inside each box represents the median value. Each box plot represents the range between the 25th and 75th quartile within the data; the cap on each end represents data within 1.5 inter-quartile ranges. Any data outside this range is visualized by individual dots. For both IMRT and VMAT, the majority of the cases had a similar range of Dmax between the dual-layer and TB MLC plans with one outlier. The dual-layer MLC in general has a slightly smaller range of Dmax distribution. When comparing across planning techniques, generally VMAT saw higher Dmax than IMRT for both the dual-layer and TB MLC plans.

Another comparison shown in Figure 1 is the total number of MUs required to deliver the treatment plans. In general, the dual-layer MLC delivery system requires higher total MU, which is expected because the Halcyon uses 6FFF energy compared to the 6 MV flattened beam for head and neck plans on the Truebeam platform. The inherently lower effective energy of the 6FFF beam, as well as the non-flat beam profiles, would require more MUs to deliver the same dose to a given depth.

**Figure 2:**
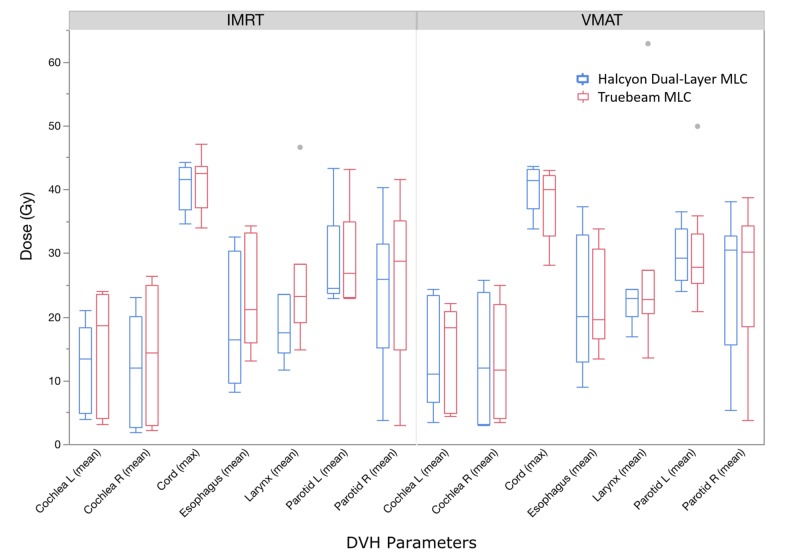
Box plots comparing key dose-volume histogram (DVH) parameters of organs-at-risk (OARs) for two delivery systems and intensity-modulated radiation therapy (IMRT) or volumetrically modulated arc therapy (VMAT) techniques. Red represents Truebeam® plans using TB multi-leaf collimator (MLC), and blue represents Halcyon™ plans using the Halcyon™ dual-layer MLC.

OAR doses were compared for key dose-volume histogram (DVH) parameters and is visualized in Figure 2. It is evident that for the majority of the DVH parameters, TB and dual-layer MLC plans have a large overlap of boxes, indicating the similarity in range of the underlying distribution of data. This is especially true for VMAT plans.

**Figure 3:**
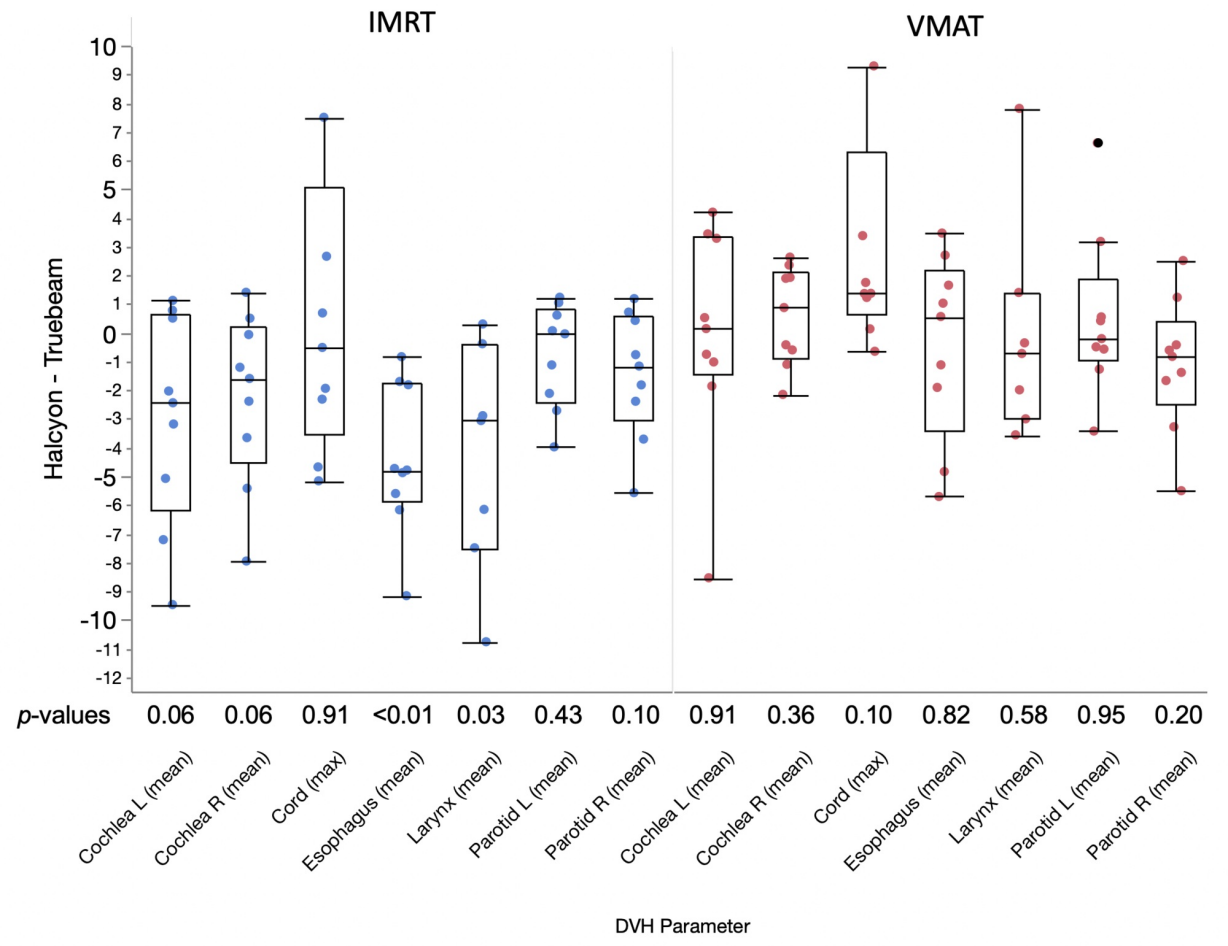
Box plots, distributions, and Wilcoxon signed-rank test p-values of the paired differences in organs-at-risk (OARs) dose-volume histogram (DVH) parameters between paired plans (Truebeam® multi-leaf collimator (TB MLC) and Halcyon™ dual-layer MLC) for the same patients. A negative number in the vertical axis means that Halcyon™ has lower OAR dose than Truebeam, i.e. better performance.

The paired differences between TB MLC and the dual-layer MLC plans are highlighted in Figure 3. Comparing IMRT and VMAT techniques, the differences in OAR dosimetric parameters for VMAT were mostly centered around zero except for the maximum cord dose, indicating similar performances between TB MLC and the dual-layer MLC for VMAT plans. For cord Dmax, all plans had Dmax < 48 Gy per RTOG 0522. The median difference between dual-layer and TB MLC on cord Dmax is 1.48 Gy. For IMRT plans, the difference between dual-layer MLC and TB MLC plans was mostly distributed in the negative region, which indicates that the dual-layer MLC in general achieved better OAR sparing when compared to TB MLC for IMRT plans.

Wilcoxon signed-rank tests were performed between TB and dual-layer MLC plan pairs to determine the statistical significance of the observed differences, and the resulting p-values were included in Figure 3. Overall no statistical significance was observed for VMAT plans, except for the maximum cord dose: a lower maximum cord dose was observed for VMAT plans using TB MLC. However, from Figure 2 it is evident that all plans had cord Dmax < 45 Gy, which is our clinical limit. For IMRT plans, reductions in OAR dose were statistically significant (p < 0.05) for mean doses for esophagus, larynx, and marginal (p = 0.06) for left and right cochlea. Lower mean dose was observed for both parotid glands based on distribution of points but was not significant statistically. These results agreed with the general observation from Figure 2.

Plan delivery time comparison

**Figure 4:**
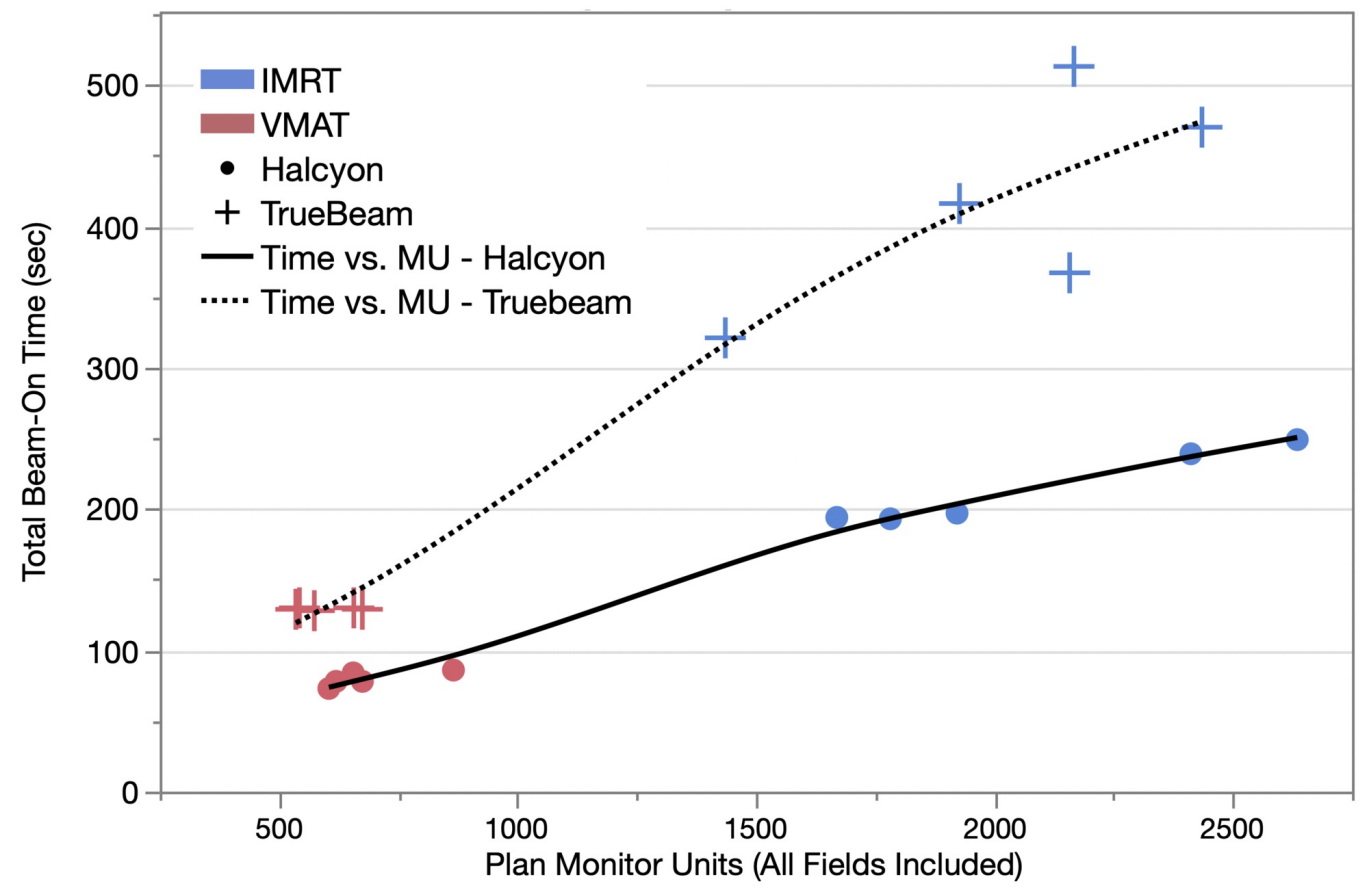
Plot of total beam-on time for plan delivery vs. plan total monitor units (MU) for the two techniques and two delivery platforms. Blue corresponds to intensity-modulated radiation therapy (IMRT) plans, and red to volumetrically modulated arc therapy (VMAT) plans. Halcyon™ and Truebeam® plans are represented by circle and cross markers, respectively. A trend line was also fitted for the two platforms (Halcyon™ and Truebeam) using combined data points from both IMRT and VMAT plans.

Plan delivery time was evaluated and shown in Figure 4. Overall the Halcyon platform was able to deliver treatment for the same case with substantially reduced time: on average a 42.8 ± 8.5% reduction from the corresponding Truebeam plans was observed despite the higher total MU as shown in Figure 1. Average reduction in beam-on time brought on by the Halcyon platform was 3.4 ± 1.2 min for the IMRT technique and 0.8 ± 0.1 min for the VMAT technique. This suggests that the Halcyon platform can offer nearly double the delivery efficiency when compared to the Truebeam platform in the real-world scenario.

Another observation that can be made from Figure 4 is that for VMAT plans in the low MU range, the Truebeam platform had nearly the same delivery time regardless of MU while Halcyon still maintains a relatively linear relationship between beam-on time and total MU. This is mainly because at this MU range the Truebeam delivery is mainly limited by the gantry angle rotation speed (six degree per sec or one revolution per min) therefore lowering the total MU will not translate into saving time during delivery. For Halcyon, however, since the gantry rotation can be four times faster, delivery efficiency is no longer limited by the gantry rotation speed, and allows the user to reduce delivery time by limiting total MU if desired. Therefore generally speaking, the delivery speed of the current Halcyon design is limited by mainly the maximal dose rate (800 MU/min).

MLC parameters impact on OAR doses

In this section, four types of hypothetical TB MLCs were simulated with DLG, leaf speed, and transmission, and all three of the above matched to the specifications of the Halcyon dual-layer MLC. Treatment plans were then re-optimized using these simulated MLC systems that resemble one or three parameters of the dual-layer MLC features.

**Figure 5:**
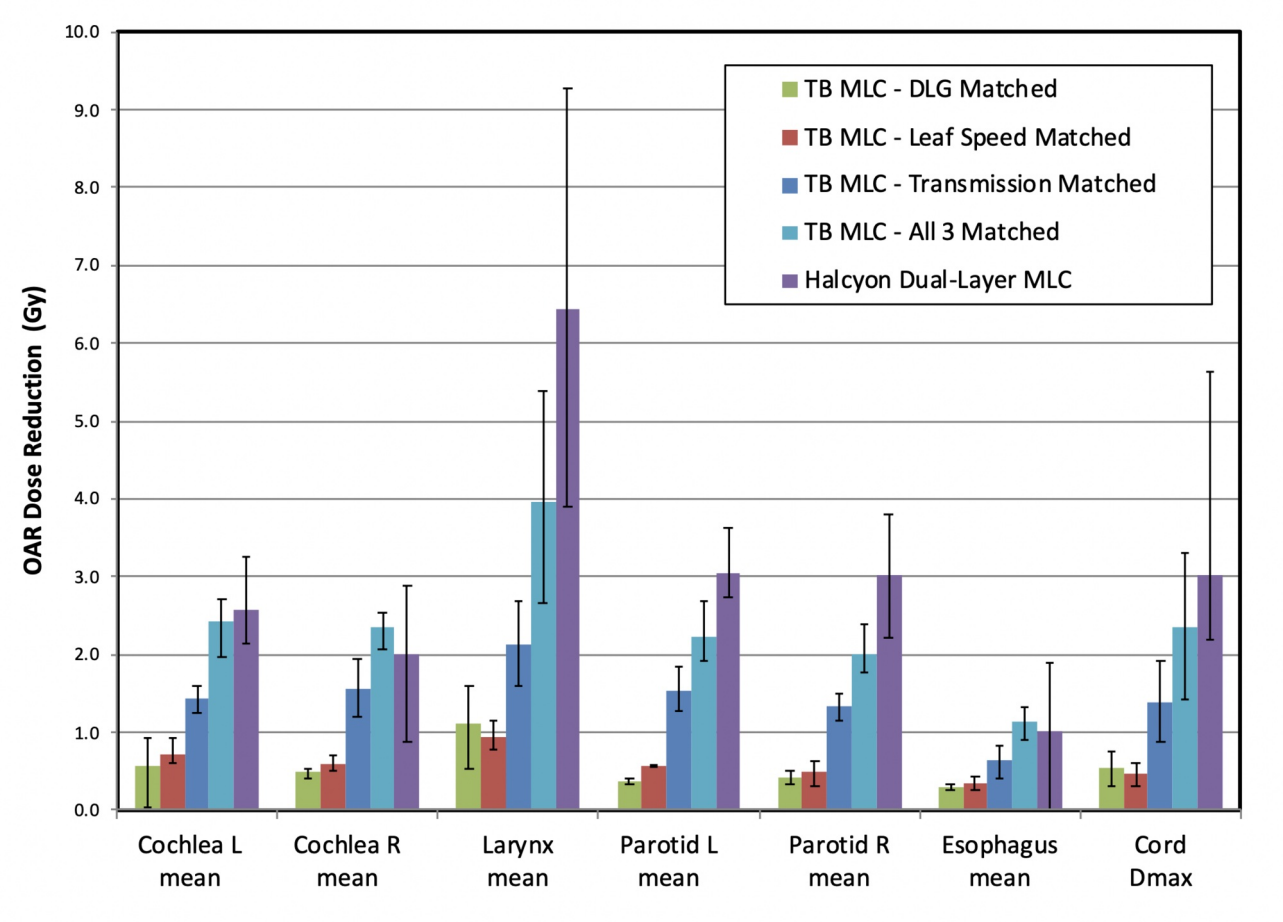
Organs-at-risk (OARs) dose reduction observed in plans using different simulated Truebeam® multi-leaf collimator (TB MLC) with key parameters matched to those of the Halcyon™ dual-layer MLC. Reference OAR doses, i.e. zero reduction, were set to plans done with actual TB MLC. Error bars indicate the full range of all test cases for a particular dose-volume histogram (DVH) parameter.

OAR dose reduction from simulated MLCs and the actual dual-layer MLC was extracted from re-optimized plans and visualized in Figure 5. At a glance, all simulated MLCs improved OAR doses for the cases analyzed. This is expected since the dual-layer MLC features improved DLG, leaf speed, and transmission when compared to TB MLC, and therefore would translate into improved dosimetric performance. When comparing plans with only one MLC parameter matched to the dual-layer MLC, it is evident that matching MLC transmission produced the closest OAR dose reduction compared to the reduction achieved with the actual dual-layer MLC. This means that MLC leakage is the dominating factor that improved the quality of the plan. This is consistent with the finding from Topolnjak et al. [9].

## Discussion

In this study we first compared Halcyon’s dual layer MLC’s performance in IMRT and VMAT treatment for head and neck cancer to that of Truebeam’s Millennium 120 MLC in terms of plan quality and delivery speed, and later identified which MLC parameter contributed the most to observed plan quality differences. Our finding on VMAT plan quality and delivery time comparison between Halcyon and Truebeam agrees with Michiels et al., who evaluated head and neck patients and concluded that Halcyon maintains plan quality for VMAT technique [10]. The main contribution and added value from our study to the current knowledge of Halcyon delivery system are the differences in IMRT plan quality and delivery time, and investigations on MLC parameter’s impact on observed plan quality differences for IMRT plans.

When comparing dual-layer MLC with TB MLC on OAR doses for IMRT plans, one could question whether Figure 3 and Figure 5 showed meaningful differences: only some OAR doses were significantly improved but all OAR doses were consistently improved using dual-layer MLC, which is shown in Figure 5. This inconsistency is likely due to the difference in study aims and designs, as well as sample differences. In the planning study, the planners were instructed to approach each case as if it were a new clinical case, i.e., to freely adjust planning objectives until they think that it is the best achievable plan in a clinically relevant timeframe. This study design was based on the hypothesis that Halcyon plans using 6FFF beams are comparable to Truebeam plans using 6 MV beams, which is our clinical standard. However, for the MLC parameters study, focus was on isolating OAR dose difference as a result of changes in MLC parameters by keeping the same beam energy, beam arrangement, and planning objectives across all treatment plans. In addition, Figure 3 included two unilateral cases that were not included in Figure 5, which might also have contributed to the differences in observed OAR dose reduction. Therefore, the results from Figure 3 and Figure 5 should not be directly compared due to the differences in study design and sampling.

It was observed that the OAR dose reduction is more pronounced for IMRT plans than for VMAT plans. This is mainly due to higher MU used in IMRT to deliver same target dose compared to VMAT. Since target dose is the same, for a given MLC transmission factor higher MU results in more leakage radiation through the MLC reaching the OARs. Also because leakage radiation through the MLC is proportional to both the transmission factor and total MU, for a given transmission factor reduction, e.g. from TB to Halcyon MLC, more MU would result in more pronounced difference in the transmitted radiation beyond MLC, which directly contributes to OAR dose. This explains why the difference in OAR dose due to MLC transmission change is more pronounced for IMRT than for VMAT.

It is evident from Figure 5 that even with all three parameters, transmission, leaf speed, and DLG, matched to the Halcyon dual-layer MLC, there are still residual differences when compared to the actual dual-layer MLC. This residual difference is likely to be a result of two competing factors that cannot be easily modeled by changing the TB MLC's parameters. Firstly, the dual-layer MLC has 1 cm width, whereas TB MLC has 0.5 cm width. Even though statistical differences were not observed in most of the dosimetric parameters from the planning study presented in the first part of this paper, the effect of MLC width may still have an impact on normal tissue dose, particularly for small structures like the cochlea. In some cases, limited sparing power due to wider leaves might overshadow the gain from other improvements, resulting in inferior DVH parameters for some structures.

On the other hand, because Halcyon MLCs are dual-layer stacked, the area under two layers of MLCs effectively experiences even less leaf leakage. The leakage with two layers blocking the beam can be approximated to be 0.007^2^ = 0.000049, or ~0.005%. This further reduces leakage and transmission dose to blocked areas, making the actual dual-layer MLC perform better than a single layer TB MLC with 0.007 transmission factor. There are other factors that were not simulated, such as the full range modulation and interdigitation, which are limited in TB MLC model. The ability of unrestricted leaf movement allows for additional capability to modulate beam intensity. This could explain why in some cases, the dual-layer MLC has lower mean normal tissue dose compared to simulated TB MLC, even with wider leaves. For Truebeam linacs with jaw-tracking capabilities, the dynamic jaws conforming to MLC openings may help reduce the MLC leakage to blocked regions albeit to a limited level since the jaws can only conform to the least extended (i.e., most open) leaf position for any given aperture.

It should be noted that Halcyon MLC transmission factor used in this study was based on pre-clinical system that we had access to at the time of data acquisition. In the clinical release of Eclipse™ treatment planning system the transmission factor was changed to 0.047%. This will likely enhance the normal tissue sparing effect that was observed due to transmission factor being smaller than TB MLC.

## Conclusions

When compared to Truebeam's Millennium 120 MLC, the Halcyon treatment delivery system's newly designed dual-layer MLC is capable of generating head and neck IMRT/VMAT plans of similar quality despite having thicker MLC leaves. For some OARs, the IMRT plan using the dual-layer MLC provided better sparing in terms of mean dose. Detailed analysis showed that this improvement was largely due to a combination of lowered leaf transmission, faster leaf speed, and smaller DLG. Among these three MLC parameters, lower leaf transmission had the largest contribution for the improved OAR sparing. Thicker MLC leaves provided stronger mechanical construction, which resulted faster MLC movement speed. The delivery speed of head and neck treatment plans using Halcyon platform were nearly twice as fast than the TB MLC for both fixed gantry beam arrangement (IMRT mode) and VMAT mode.
